# Discovery of Several Novel Targets that Enhance β-Carotene Production in *Saccharomyces cerevisiae*

**DOI:** 10.3389/fmicb.2017.01116

**Published:** 2017-06-15

**Authors:** Jia Li, Jia Shen, Zhiqiang Sun, Jing Li, Changfu Li, Xiaohua Li, Yansheng Zhang

**Affiliations:** ^1^CAS Key Laboratory of Plant Germplasm Enhancement and Specialty Agriculture, Wuhan Botanical Garden, Chinese Academy of SciencesWuhan, China; ^2^University of Chinese Academy of SciencesBeijing, China

**Keywords:** β-carotene biosynthesis, Did2, colony-color screening, novel amplifying targets, *Saccharomyces cerevisiae*

## Abstract

β-Carotene is the precursor of vitamin A, and also exhibits multiple pharmaceutical functions by itself. In comparison to chemical synthesis, the production of β-carotene in microbes by metabolic engineering strategy is relatively inexpensive. Identifying genes enhancing β-carotene production in microbes is important for engineering a strain of producing higher yields of β-carotene. Most of previous efforts in identifying the gene targets have focused on the isoprenoid pathway where the β-carotene biosynthesis belongs. However, due to the complex interactions between metabolic fluxes, seemingly irrelevant genes that are outside the isoprenoid pathway might also affect β-carotene biosynthesis. To this end, here we provided an example that several novel gene targets, which are outside the isoprenoid pathway, have improving effects on β-carotene synthesis in yeast cells, when they were over-expressed. Among these targets, the class E protein of the vacuolar protein-sorting pathway (Did2) led to the highest improvement in β-carotene yields, which was 2.1-fold to that of the corresponding control. This improvement was further explained by the observation that the overexpression of the *DID2* gene generally boosted the transcriptions of β-carotene pathway genes. The mechanism by which the other targets improve the production of β-carotene is discussed.

## Introduction

**FIGURE 1:**
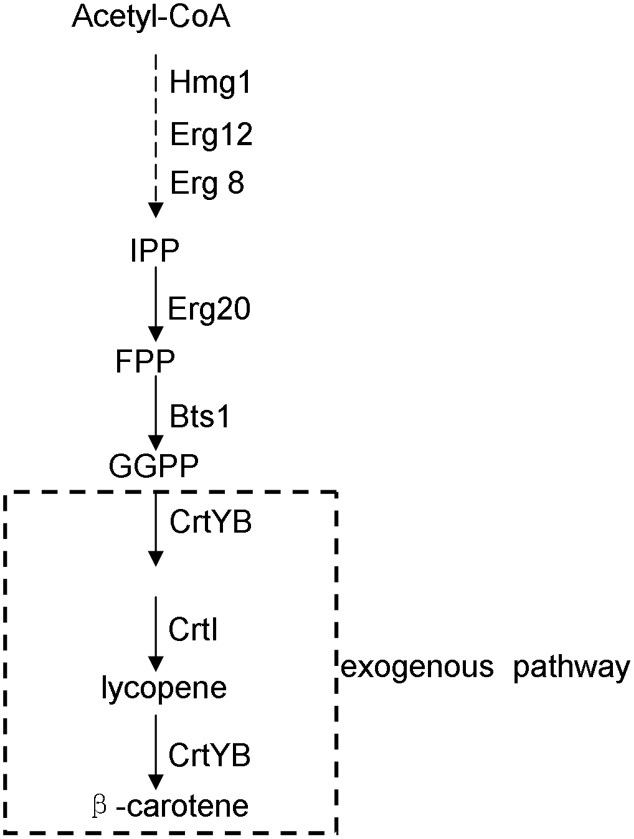
Biosynthetic pathway for β-carotene production in the engineered yeast strain of this study. Hmg1, 3-hydroxy-3-methylglutaryl coenzyme-A reductase; Erg12, mevalonate kinase; Erg20, farnesyl pyrophosphate synthase; Erg8, phosphomevalonate kinase; Bts1, geranylgeranyl pyrophosphate synthase; CrtYB, phytoene synthase and cyclase; CrtI, phytoene desaturase. The dotted lines indicate the pathway steps that were integrated into the yeast genome by this study.

β-Carotene is a carotenoid compound with multiple physiological and pharmaceutical functions: e.g., it functions in photosynthesis as a light-harvesting pigment in naturally carotenoid-producing organisms such as higher plants and photosynthetic microorganisms (Yamano et al., 1994), and has been applied as a natural pigmentation ingredient in food industry; moreover, for humans, β-carotene is the precursor of vitamin A and was ever proposed for cancer treatments (Giovannucci et al., 1995). Although β-carotene is now supplied mainly by chemical synthesis, there has been much interest in engineering the synthesis of this compound in microbes, such as the fungus *Neurospora crassa* (Armstrong and Hearst, 1996), the yeast *Candida utilis* (Miura et al., 1998), the yeast *Saccharomyces cerevisiae* (Yamano et al., 1994; Verwaal et al., 2007; Yan et al., 2012; Li et al., 2013; Xie et al., 2014), and *Escherichia coli* (Yoon et al., 2009; Zhao et al., 2013; Yang and Guo, 2014). Given the safety of the downstream applications and the convenience of genetic manipulations, the food yeast *S. cerevisiae* is becoming a desirable platform for the production of β-carotene. In *S. cerevisiae*, its native isoprenoid pathway synthesizes farnesyl pyrophosphate which can serve as the precursor of the introduced carotenoid pathway. The pathway for engineering β-carotene biosynthesis in *S. cerevisiae* is shown in **Figure 1**.

So far, there are a few metabolic engineering efforts that have been reported to enhance carotenoid productivity in *S. cerevisiae*. Those efforts mostly focused on manipulating the isoprenoid pathway that is directly connected with carotenoid formation (Yan et al., 2012; Li et al., 2013). However, the interaction between metabolic fluxes of *S. cerevisiae* confers a complex regulatory network and seemingly irrelevant genes might exert significant effects on the carotenoid pathway established in yeast by genetic engineering. For example, through adaptive laboratory evolution experiments, several genes that are relevant to lipid biosynthesis had been identified to promote carotenoid biosynthesis in *S. cerevisiae* (Reyes et al., 2014); on the other hand, deletions of several novel gene targets that are not directly associated with the isoprenoid pathway significantly promoted carotenoid formation (Ozaydin et al., 2013). The objective of this work is to discover novel amplification gene targets for improving β-carotene production in *S. cerevisiae*. A laboratory yeast strain WAT11 (Urban et al., 1997) was used as a host to construct the recombinant β-carotene-producing strain by integrating the carotenoid pathway genes derived from *Xanthophyllomyces dendrorhous* into its genome. The resultant β-carotene-producing strain was then transformed with a *S. cerevisiae* cDNA library. By screening colony color followed by measuring β-carotene yield, we reported here several novel amplification targets that significantly increased β-carotene production in *S. cerevisiae*. Among these targets, the class E protein of the vacuolar protein-sorting pathway (Did2) led to the highest improvement. The highest β-carotene yield here with the *DID2* overexpression was 5.9 ± 0.1 mg/g, which was 2.1-fold to that of the corresponding control. The mechanism for increasing the β-carotene yield by the *DID2* expression was further investigated. Our data indicated that the *DID2* overexpression up-regulated the transcription of carotenoid pathway genes, which may cause the improvement of β-carotene biosynthesis in *S. cerevisiae*.

## Materials and Methods

### Strains and Culture Conditions

All the nucleotide primers for amplifying genes in this study are listed in Supplementary Table 1. The *BTS1* (*S. cerevisiae* geranylgeranyl pyrophosphate synthase gene, GenBank accession no. NM_001183883) expression cassette, ‘*pTEF-BTS1-CYC1t*,’ was amplified by linking the *BTS1* open reading frame (ORF) with the *TEF* promoter (GenBank accession no. EF210199) and *CYC1* terminator (GenBank accession no. DQ232604) using primers 1–6 (Supplementary Table 1). The DNA cassette ‘*pTEF-BTS1-CYC1t*’ was then introduced into an integrative yeast expression vector YIplac128 at *Bam*HI/*Sal*I sites, resulting in the constructs YIplac128-BTS1. YIplac128-BTS1 was linearized with an *Eco*RV digestion and integrated into the *LEU2* locus of the WAT11/pRS406W strain, which is a carotene-producing yeast previously prepared by Li et al. (2013). The resultant yeast strain bearing the *BTS1* expression cassette was designated WYIB in this study. Yeast cells were grown on appropriate dropout medium supplemented with 2% glucose or 2% galactose as the carbon source. For monitoring the growth property of the recombinant yeast strains, single colonies were picked from plates, inoculated into appropriate liquid medium containing 2% glucose, and grown overnight at 30°C. The yeast cells were then washed with sterile water and sub-cultured into a fresh induction medium at an OD_600_ of 0.1, where 2% glucose was replaced with 2% galactose. Starting from the sub-culture, the OD_600_ of the cell cultures were measured every 24 h and the cell biomass was recorded by weighing lyophilized cell weight.

### Screening the Yeast cDNA Library by Examining Colony Color Phenotype

The λTRP-*S. cerevisiae* cDNA library, which was generated by *cre*-*lox* recombination from λTRP (Elledge et al., 1991), was ordered from the American Type Culture Collection (ATCC no. 87277). In this library, individual yeast cDNAs were inserted between *GAL1* promoter and *CYC1* terminator in the pTRP vector with *TRP1* as a selection marker. To screen the gene targets that potentially improve β-carotene biosynthesis, 10 μg of the yeast cDNAs was transformed into the WYIB strain and grown on the selective plates (dropout medium lacking uracil, leucine, and tryptophan, and containing 2% galactose and 2% agar) for colony color visual screening. The WYIB strain transformed with the empty vector pESC-TRP (Stratagene, La Jolla, CA, United States) served as a control. After being grown for 7 days, the colony color phenotype was suitable for visibly validation. Three rounds of the colony color screening were applied: First, the colonies harboring the *S. cerevisiae* cDNA library were compared against the control one on same plates and the colonies with increased orange color were selected; second, the selected colonies were re-spotted onto plates and the truly colonies with increased orange color relative to the control were used for the next screening; third, the plasmids containing the *S. cerevisiae* cDNAs were isolated from the selected colonies and re-transferred into the WYIB strain to confirm the color phenotype compared with the control strain. After the three rounds of selections, colonies with increased orange color were eventually considered as the positive clones and their contained cDNA inserts were discovered through plasmid isolation followed by sequencing.

### Screening the Gene Targets by Measuring β-Carotene Yield

To examine whether the above positive cDNA inserts truly enhance β-carotene production in *S. cerevisiae*, the ORFs of the cDNA candidates were amplified using primers 7–18 and individually inserted into the yeast expression vector pESC-TRP under the control of a galactose inducible promoter, yielding the constructs of pESC-TRP-BMH1, pESC-TRP-CAR1, pESC-TRP-DID2, pESC-TRP-PDC5, pESC-TRP-TIF5, and pESC-TRP-VOA1. The resultant constructs were then separately transformed into the WYIB strain to give the strains of WYIB-BMH1, WYIB-CAR1, WYIB-DID2, WYIB-PDC5, WYIB-TIF5, and WYIB-VOA1, respectively. The WYIB strain transformed with the empty vector pESC-TRP served as the control and designated WYIB-TRP. Single colonies of each transformant were cultured in dropout liquid medium as described above, and the 72 h-grown cells at stationary phase were harvested for measuring β-carotene yield. Two-tailed *t*-test was performed for the statistical analysis. To further confirm the increment in β-carotene production by the best target (DiD2), the strain WYIB-DID2 was compared with the control strain WYIB-TRP in a time course with respect to the ability of producing β-carotene.

To verify the universality of the improving role of *DID2* on β-carotene biosynthesis, another yeast strain with a genetic background distinct from the WAT11 strain used above, namely CEN.PK2-1C, was used as a host to test the *DID2* effect. The carotenoid-producing vectors, pRS406W and plac128-BTS1, were linearized with *Stu*I and *Eco*RV, respectively, and simultaneously integrated into the genome of CEN.PK2-1C under the *URA3* and *LEU2* locus, yielding the carotene-producing strain of CYIB. To test the *DID2* effect under the CYIB background, the constructs of pESC-TRP-DID2 and pESC-TRP were separately transformed into the CYIB strain to give the strains of CYIB-DID2 and CYIB-TRP, then, the β-carotene yields by both strains were compared in a time course.

### β-Carotene Extraction and Analysis

The β-carotene was extracted from the yeast cultures with acetone/0.2% pyrogallol in methanol (w/v) (80:20, v/v) as described previously (Li et al., 2013), and its yields were quantified by high performance liquid chromatography (HPLC) analysis. The HPLC analysis was performed on an LC-20AT instrument (Shimadzu, Kyoto, Japan) with an inertsil ODS-SP reversed-phase column (4.6 mm × 250 mm, 5 μm). Metabolites were separated at 2 mL/min and 25°C in acetonitrile/methanol (v/v = 50:50), and monitored at a wave length of 450 nm. The β-carotene production was quantified using a standard calibration curve which was obtained by authentic β-carotene from Sigma Chemical Co. (Cat. no. 7235407) in five gradient concentrations.

### Quantitative Reverse Transcription-Polymerase Chain Reactions (qRT-PCRs)

Total RNA was isolated from the yeast cells using an EASYspin RNA kit (Aidlab, Beijing, China). RNA was treated with DNase I (Thermo Scientific) to remove genomic DNA contaminations, and reversed to cDNA using Oligo-dT18 primer (Qsingke, Wuhan, China) and M-MLV reverse transcriptase (Thermo Scientific) following the provided protocols. To analyze the transcript levels of genes encoding 3-hydroxy-3-methylglutaryl coenzyme-A reductase (*HMG1*, GenBank accession no. NM_001182434), mevalonate kinase (*ERG12*, GenBank accession no. NM_001182715), phosphomevalonate kinase (*ERG8*, GenBank accession no. NM_001182727), farnesyl pyrophosphate synthase (*ERG20*, GenBank accession no. NM_001181600), geranylgeranyl pyrophosphate synthase (*BTS1*, GenBank accession no. NM_001183883), phytoene synthase and cyclase (*crtYB*, GenBank accession no. AY177204) and phytoene desaturase (*crtI*, GenBank accession no. AY177424), specific primers 19–32 (Supplementary Table 1) were used to amplify their transcripts. To normalize the variation between the cDNA preparations, the *S. cerevisiae* actin gene (GenBank accession no. NM_001179927) was used as an internal standard and amplified with the primers 33/34. Real-time RT-PCRs were performed in three biological replicates with three technical repeats, using FastStart Universal SYBR Green Master mix (Rox) (Roche, Mannheim, Germany) on an ABI 7500 FAST Real Time PCR Systems (Life Technologies, United States). The relative expression was estimated using the comparative threshold cycle method (Schmittgen and Livak, 2008). Two-tailed *t*-test was performed to evaluate the gene expression difference between the control strain WYIB-TRP and the strain WYIB-DID2.

## Results

### Screening Novel Targets for High β-Carotene Titers in Yeast

**FIGURE 2:**
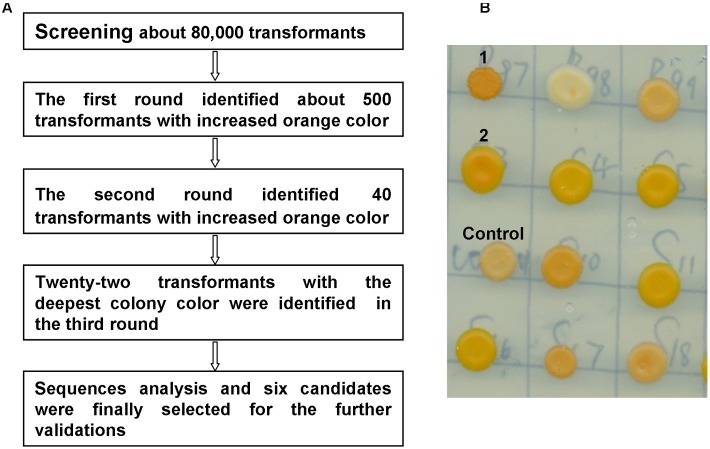
Colony color-screening identified the *S. cerevisiae* transformants that accumulated higher yields of β-carotene. **(A)** The scheme for the colony-color screening; **(B)** a representative picture showing the color-screening on plates, for example, in this figure, the transformants 1 and 2 showed the increased orange color in comparison with the control colony.

To identify the genes that potentially improve β-carotene production, the carotenoid-producing strain WYIB was screened with the collection of the yeast cDNAs (ATCC no. 87277). Transformants with a higher production of β-carotene were visually sieved by the increased orange color. Previously, this approach was successful in quickly identifying gene targets that affect carotenoid biosynthesis (Ozaydin et al., 2013). In the first round of the screening, 500 transformants among those showing the increased orange color out of about 80,000 colonies were selected. The colonies selected from the primary screening were then subjected to additional two rounds of the color validations. Eventually, there were twenty-two colonies with the deepest orange color identified from the color screening (**Figure 2**) The cDNA inserts of these 22 colonies were then discovered by sequencing. Each of the colonies was found to bear only one cDNA insert. Among these, there were five gene targets that encode ribonucleoproteins (RNPs) and nine genes that were present in an anti-sense form. By a blast search, the remaining eight cDNA inserts were found to encode enzymes of glyceraldehyde-3-phosphate dehydrogenase (Tdh1), pyruvate kinase (Cdc19), arginase (Car1), class E protein of the vacuolar protein-sorting pathway (Did2), pyruvate decarboxylase (Pdc5), 14-3-3 protein (Bmh1), translation initiation factor (Tif 5), and vacuolar H(+)-ATPase subunit 1 (Voa1), respectively. Tdh1 and Cdc19 are the enzymes required for glycolysis process which is upstream of the isoprenoid pathway and which also generate acetyl-CoA, the precursor of the carotenoid biosynthesis. Because this study focused on the genes outside the isoprenoid pathway, the Tdh1 and Cdc19 were not further investigated and the remaining six candidates were in the further validations.

**FIGURE 3:**
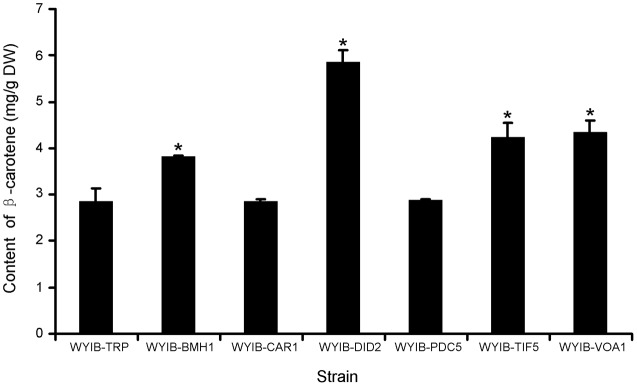
Production of β-carotene by the recombinant *S. cerevisiae* strains. The experiment was performed in three biological replicates. WYIB, the WAT11 yeast strain integrated with the β-carotene biosynthetic pathway; the WYIB strain was transformed with the empty vector pESC-TRP to give the control strain WYIB-TRP, and transformed with the gene targets to generate the recombinant yeast strains (WYIB-BMH1, WYIB-CAR1, WYIB-DID2, WYIB-PDC5, WYIB-TIF5, and WYIB-VOA1). The products of the gene targets shown in the yeast strains are 14-3-3 protein (Bmh1), arginase (Car1), class E protein of the vacuolar protein-sorting pathway (Did2), pyruvate decarboxylase (Pdc5), translation initiation factor (Tif5), and vacuolar H(+)-ATPase subunit 1 (Voa1). Asterisks indicate significant differences. *P* < 0.05.

**FIGURE 4:**
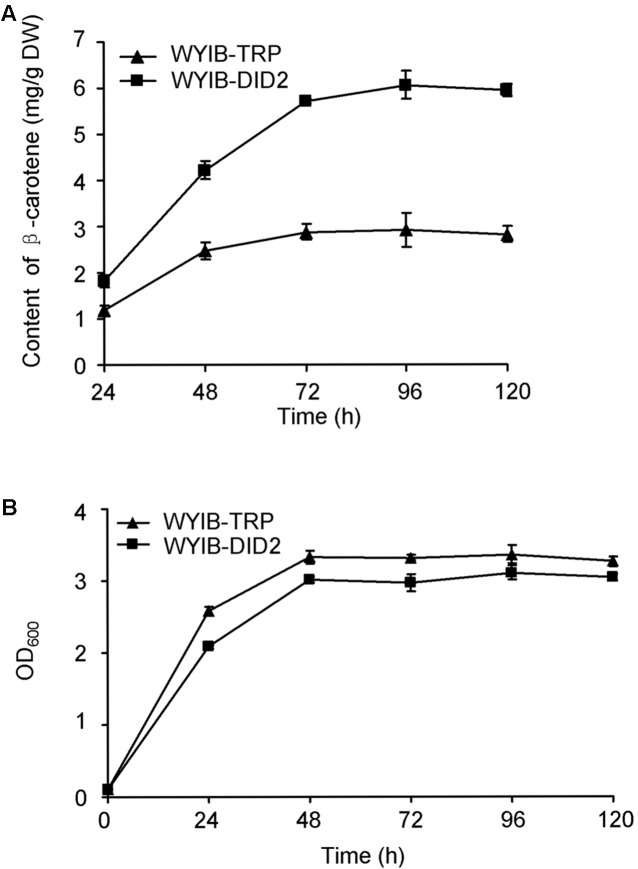
The production of β-carotene by the WYIB-TRP and WYIB-DID2 strains **(A)** and their growth curves **(B)** in a time course. The measurement was performed in three biological replicates.

In order to ensure their positive effects of the above six candidates (*CAR1, DID2, PDC5, BMH1, TIF5*, and *VOA1*) on the β-carotene production in *S. cerevisiae*, their ORFs were individually re-amplified, cloned into the yeast expression vector pESC-TRP, and transformed into the WYIB strain. As a control, the WYIB strain was transformed with the empty vector pESC-TRP. The β-carotene yield accumulated by these transformants was measured by HPLC analysis. Compared with the control, the expressions of *DID2, VOA1, TIF5*, and *BMH1* increased the β-carotene production to 205, 152, 148, and 133%, respectively, whereas no significant increment was observed by over-expressing either *PDC5* or *CAR1* (**Figure 3**). Among these targets, the *DID2* over-expression led to the largest increase in β-carotene levels. The enhanced β-carotene production in the *DID2* over-expressing strain (WYIB-DID2) was further assessed in a time course compared to the control strain (WYIB-TRP). The strain WYIB-DID2 produced significantly higher levels of β-carotene than the control strain at all the time points (**Figure 4A**). The β-carotene level accumulated by the strain WYIB-DID2 at the 120 h-time point was 5.9 ± 0.1 mg g^-1^ dry cell weight, which was 2.1-fold of that of the control strain (2.8 ± 0.2 mg g^-1^ DW). The growth property of the strain WYIB-DID2 was measured as well. As shown in **Figure 4B**, except for a little bit reduction, the growth profile of WYIB-DID2 was comparable to that of the control strain, indicating that the up-regulation of *DID2* exerted no serious inhibition on yeast growth.

### The *DID2* Improving Effect on β-Carotene Biosynthesis is Universal in Yeast

To test the universality of the *DID2* effect, we integrated the carotenoid pathway into another yeast strain CEN.PK2-1C and expressed the *DID2* gene under this yeast background. The production of β-carotene was compared in a time-course manner between the strain containing the *DID2* (CYIB-DID2) and the control strain harboring the empty vector pESC-TRP (CYIB-TRP). As shown in Supplementary Figure 1A, in comparison with the control, the *DID2* expression increased the β-carotene yield at all the time points, and the levels of β-carotene produced by CYIB-DID2 were 1.8- to 2.4-fold of those produced by the control strain CYIB-TRP. Moreover, once again, the *DID2* expression did not significantly inhibit the yeast growth of the CEN.PK2-1C strain (Supplementary Figure 1B).

### The Target *DID2* Induced Higher Expressions of β-Carotene Biosynthetic Genes

**FIGURE 5:**
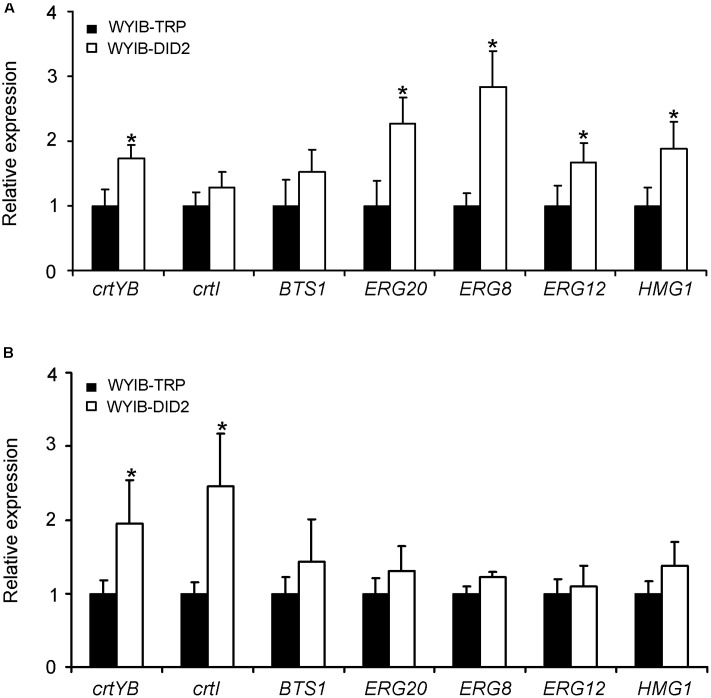
The effects of the *DID2* overexpression on the transcriptions of *crtYB, crtI, BTS1, ERG8, ERG20, Erg12*, and *HMG1* at either exponential (24 h-culture) **(A)** or stationary phase (72 h-culture) **(B)**. The *S. cerevisiae* actin gene (GenBank accession no. NM_001179927) served as an internal standard to normalize the cDNA variations. The data represent the average and standard deviation of three biological repeats. Asterisks indicate significant differences. *P* < 0.05.

To explain why the *DID2* over-expression improve the production of β-carotene in *S. cerevisiae*, we tested the effects of the *DID2* over-expression on the expression levels of the seven β-carotene biosynthetic genes (*HMG1, ERG12, ERG20, ERG8, BTS1, crtYB*, and *crtI*) (**Figure 1**). Transcript abundances of these genes were measured at either the exponential (24 h-culture) or stationary phase (72 h-culture). Compared to those in the control yeast cells (WYIB-TRP), the expression levels of these genes in the *DID2*-overexpressed yeast cells (WYIB-DID2) generally increased to different extents at both growth stages (**Figure 5**). For instance, at the exponential phase, the transcript levels of *HMG1, ERG20*, and *ERG8* in the upstream pathway were up-regulated by 0.9-, 1.3-, and 1.8-fold, respectively (**Figure 5A**); at the stationary stage, the increment of the *crtYB* expression levels reached up to 2.0-fold while the *crtI* expression increased up to 2.5-fold (**Figure 5B**). Thus, it suggested that the improvement in the β-carotene production induced by *DID2* over-expression was most likely caused by the elevated transcript levels of β-carotene biosynthetic genes.

## Discussion

*Saccharomyces cerevisiae* is becoming a desirable host for β-carotene production because of its food grade property in the process. Recent modifications made *S. cerevisiae* accumulate 25 g per liter of artemisinic acid (a sesquiterpene) (Paddon et al., 2013), suggesting its big potential of producing exogenous terpenoids at a large scale. However, to the best of our knowledge, the best β-carotene yield by this system so far is less than 20 mg per gram dry cell weight. Only few previous studies reported on the improvement of carotenoid production in *S. cerevisiae* by metabolic engineerings, and most of these studies focused on the isoprenoid flux toward carotenoid production (Yan et al., 2012; Li et al., 2013). More efforts for identifying novel gene targets to enhance carotenoid productivity are required. In this study, eight gene targets were identified through screening a yeast cDNA library. Of these targets, *TDH1* and *CDC19* code for glyceraldehyde-3-phosphate dehydrogenase and pyruvate kinase, respectively, both enzymes catalyze the reactions in glycolysis process which occur in the upstream isoprenoid pathway. It is reasonable that the targets of *TDH1* and *CDC19* appeared by the colony color screening since their up-regulations might provide the increased precursors for the biosynthesis of carotenoid precursor. The rest six gene targets are outside of the isoprenoid pathway, and consequently fell into the focus of this study for further analysis. By measuring β-carotene yield, we eventually concluded that four gene targets (*DID2, BMH1, VOA1*, and *TIF5*) would increase the β-carotene production (**Figure 3**). To the best of our knowledge, this is the first report of these targets performing positive effects on carotenoid production. Voa1 is an integral membrane protein that functions in regulating the acidification of its resident organelle (Ryan et al., 2008). The increased expression of *VOA1* may result in a low pH environment in cell membranes that was previously suggested to be the accumulation sites of carotenoids (Sandmann et al., 1999). There is a report which shows that carotenoids seem to be more stable when cell membranes are acidified (Imasheva et al., 2006). Therefore, the increased *VOA1* expression may stimulate more acidified membrane conditions which are favorable for the storage of β-carotene. The target *TIF5* encodes a translation initiation factor that functions in the process of protein translations. The mechanism of enhancing β-carotene production by the *TIF5* over-expression is not clear, but its activity might be advantageous for the translations of the carotenoid pathway enzymes. The target *BMH1* codes for a yeast 14-3-3 protein which are highly conserved enzymes in a wide range of eukaryote organisms, and are involved in many cellular processes through interacting with other protein and/or regulating gene transcriptions (Trembley et al., 2014). Bmh1 protein localizes only to the cytoplasm at the steady-state conditions (Imasheva et al., 2006), but it becomes localized to the nucleus upon DNA replication stress (Tkach et al., 2012). In this study, the enhanced β-carotene production implied that Bmh1 might be trans-located into the cell nucleus at the exponent phase to act transcriptional regulations, causing higher levels of β-carotene.

The target *DID2* encodes a subunit of endosomal sorting complexes required for multivesicular body (MVB) formation in eukaryotic cells (Babst, 2005). *DID2* over-expression improves the production of β-carotene in two *S. cerevisiae* strains WAT11 and CEN.PK2-1C with different genetic background, indicating the universality of the improving role of *DID2* expression on β-carotene biosynthesis in *S. cerevisiae*. Overexpressing *DID2* led to a small reduction of yeast growth in this study (**Figure 4B** and Supplementary Figure 1B), which might be the result of attenuating growth factor signaling by the fortified MVB process through recruiting more Did2 (Katzmann et al., 2002). When the *DID2* expression was induced, the expression levels of *HMG1, ERG12, ERG20, ERG8, BTS1, crtYB*, or *crtI* were enhanced (**Figure 5**), suggesting the improvement of β-carotene production by the *DID2* was accompanied by the increased transcriptions of the pathway genes. It is currently not clear how the *DID2* expression increase the transcript levels of β-carotene biosynthetic genes in *S. cerevisiae*.

In summary, several novel amplification targets, which are outside of the isoprenoid pathway, have been found to promote β-carotene biosynthesis in *S. cerevisiae*. Among these, the best target is the *DID2* gene. Overexpression of the *DID2* gene increased the transcript levels of β-carotene biosynthetic genes, which ultimately caused a higher production of β-carotene. The gene targets identified in this study would be expected to have promoting effects on the production of other carotenoids as well.

## Author Contributions

YZ designed this study and wrote the manuscript; JaL performed the yeast transformation, β-carotene analysis and gene expression analysis; JS screened the yeast cDNA library; ZS performed gene cloning; JgL constructed the *S. cerevisiae* cDNAs library; CL and XL provided the assistance in HPLC analysis.

## Conflict of Interest Statement

The authors declare that the research was conducted in the absence of any commercial or financial relationships that could be construed as a potential conflict of interest.
