# Supplementary material for: Discovery of Several Novel Targets that Enhance β-Carotene Production in Saccharomyces cerevisiae
Source: Front Microbiol. 2017 Jun 15;8:1116. doi: 10.3389/fmicb.2017.01116 (PMC5471310; doi:10.3389/fmicb.2017.01116)
Supplement: Supplementary file 1 [file Data_Sheet_1.doc]

Supplementary Material

# Discovery of several novel targets that enhance β-carotene production in *Saccharomyces cerevisiae*

**Jia Li^1a^, Jia Shen^1a^, Zhiqiang Sun^1^, Jing Li^1^, Changfu Li^1^, Xiaohua Li^1, 2^, Yansheng Zhang^1^***

^a^ These authors contributed equally to this work.

*** Correspondence:** Yansheng Zhang: zhangys@wbgcas.cn

# Supplementary Figures and Tables

## Supplementary Figure

#

#
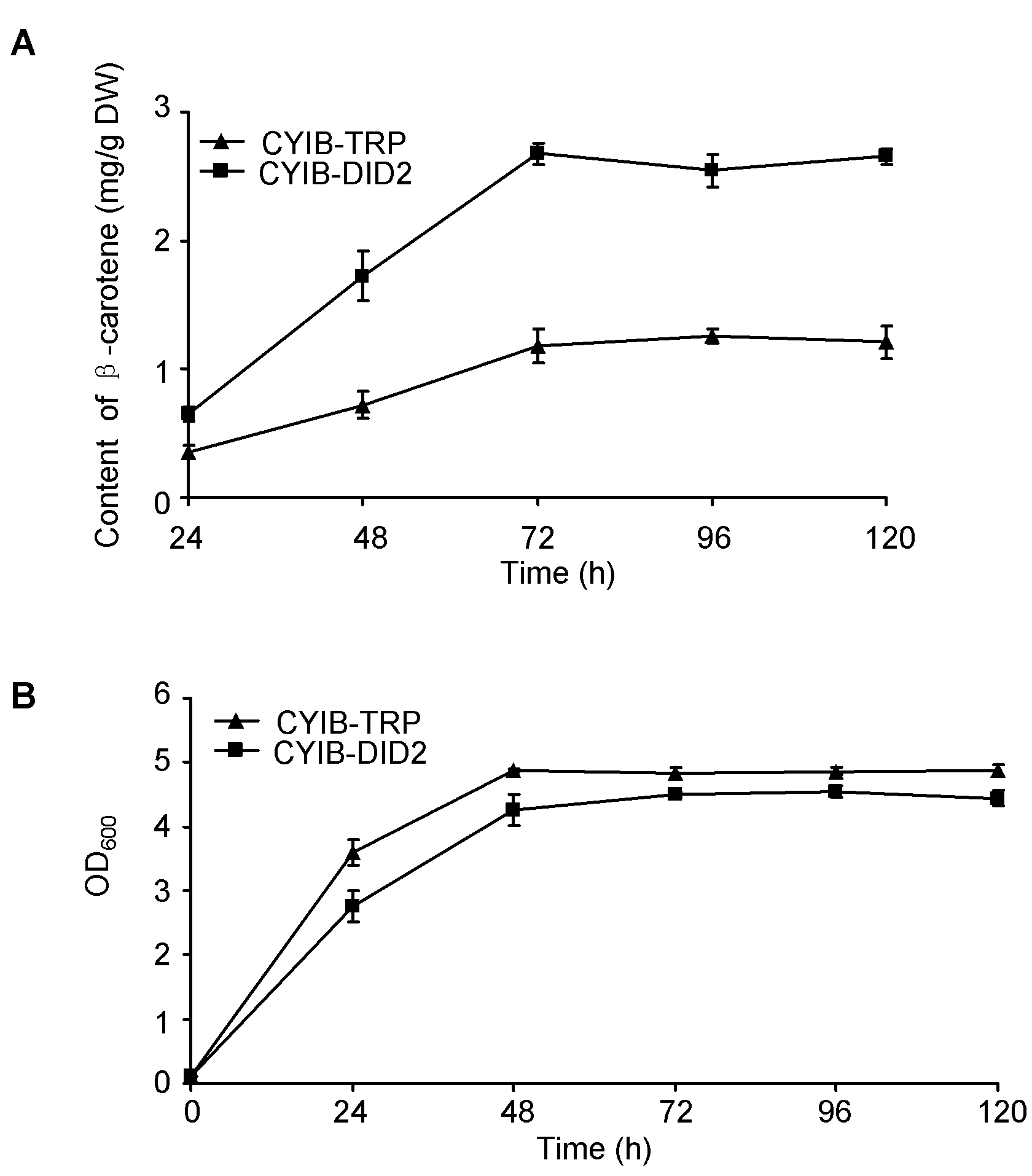


**Supplementary Figure 1. The production of β-carotene (A) and yeast growth (B) were compared between CYIB-DID2 and CYIB-TRP in a time course.** The measurement was performed in three biological replicates.

## Supplementary Table

# Supplementary Table 1. Primers used in this study

| NO | | Primers | Sequence(5’ to 3’) |
| --- | --- | --- | --- |
| For amplifying the *BTS1* expression cassette | | | |
| 1 | TEF-F | | CTGCAGATAGCTTCAAAATGTTTCTACTCC |
| 2 | TEF-R | | GCTCATCTATCTTGGCCTCCATAAACTTAGATTAGATTGCTATGC |
| 3 | BTS1-F | | GCATAGCAATCTAATCTAAGTTTATGGAGGCCAAGATAGATGAGC |
| 4 | BTS1-R | | GCGTGACATAACTAATTACATGATGTCACAATTCGGATAAGTGGTC |
| 5 | CYC1-F | | GACCACTTATCCGAATTGTGACATCATGTAATTAGTTATGTCACGC |
| 6 | CYC1-R | | GGTACCGCAAATTAAAGCCTTCGAGC |
| For amplifying the open reading frames of cDNA candidates | | | |
| 7 | BMH1-F | | CGCGGATCCATGTCAACCAGTCGTGAAG |
| 8 | BMH1-R | | CCGCTCGAGTTATTACTTTGGTGCTTCACC |
| 9 | CAR1-F | | GGATCCATGGAAACAGGACCTC |
| 10 | CAR1-R | | GTCGACCTACAATAAGGTTTCACC |
| 11 | DID2-F | | GGATCCATGTCACGTAATTCTGCAGC |
| 12 | DID2-R | | GGTACCTCAGCCCCTCAATGCTCT |
| 13 | PDC5-F | | CGCGGATCCATGTCTGAAATAACCTTAGG |
| 14 | PDC5-R | | CGCGGATCCTTATTGTTTAGCGTTAGTAGC |
| 15 | VOA1-F | | GGATCCATGGTGTTCG GTCAGCTG |
| 16 | VOA1-R | | GTCGACTTAATTGTTTTTTTTTATTGGG |
| 17 | TIF5-F | | GGATCCATGTCTATTA ATATTTGTAG |
| 18 | TIF5-R | | GTCGACCTATTCGTCGTCTTCTTC |
| For qRT-PCR | | | |
| 19 | HMG1-F | | GGTCGTGGTAAGAGTGTCGT |
| 20 | HMG1-R | | ATTAGCTGCATGTGCGTTAA |
| 21 | ERG12-F | | CTCAATCGGTGTTTCTCATCC |
| 22 | ERG12-R | | CCCACCCAAGTCTGTTTCA |
| 23 | ERG20-F | | TCTTTCTACTTGCCTGTCGC |
| 24 | ERG20-R | | AATGCCTTGTTGATTACCCA |
| 25 | ERG8-F | | CAAAACAGGGCTGGGCTCC |
| 26 | ERG8-R | | CCGCTACATCAAACCCGCTT |
| 27 | BTS1-F | | CGAAAGGTCAAACTGAGCAAC |
| 28 | BTS1-R | | GCGAAGCCAAATCAGGTAAA |
| 29 | crtYB-F | | AGGGTGGAGGCTTGGAGGT |
| 30 | crtYB-R | | CACAGGCGGTGTAATGAGGG |
| 31 | crtI-F | | TGGTATCGGTGGAATCGC |
| 32 | crtI-R | | TCTGGCAAGAGCAGCAAA |
| 33 | Actin-F | | GATTCTGAGGTTGCTGCTTT |
| 34 | Actin-R | | AGGAGTCTTTTTGACCCATAC |
